# The Use of Anesthetics and Pituitrin in Obstetrics

**Published:** 1917-05

**Authors:** L. H. Roddy

**Affiliations:** Waco, Texas


### The Use of Anesthetics and Pituitrin in Obstetrics.

BY DR. L. H. RODDY, WACO, TEXAS.

To mitigate the sufferings of the paturient woman has been the aim of accoucheurs for centuries. The ancients made use of narcotic potions and hypnotism, or suggestion; but very little advance was made until Sir James Y. Simpson made use of ether in a version and an extraction. That was in January, 1847. In the latter part of the same year, he discovered the anesthetic properties of chloroform and advised its use in labor cases; its use spread rapidly and after its use by Queen Victoria, it became popular, and the demand has rapidly increased until at the present day every pregnant woman first asks if you use chloroform before engaging you for her accoucheur.

A woman at parturition is an admirable anesthetic risk, and if the anesthetic is used with any degree of care, no untoward results would occur. But right here I want to condemn the custom of allowing one of the family to give a few drops of chloroform from time to time instead of having an assistant or at least a trained nurse under the guidance of the accoucheur. The choice of the anesthetic naturally lies with the obstetrician, but, wherever possible, it should limited to ether. Chloroform should never be used in the toxemias of pregnancy, especially those due to hepatic degenerations.

The first stage of labor should be conducted without any general anesthetic, but if the pains are unbearable, or the patient very nervous, morphine, gr. $\frac{1}{6}$, may be given. Never give morphine during the second stage.

During the second stage, when the patient is beginning to cry out with the pain, a few whiffs of ether should be given at each pain, the patient being allowed to come out between pains; but when the perineum is bulging and during the delivery of the head, the anesthetic should be pushed to the surgical degree—the patient is better managed, the soft parts yield more readily and the pains are not as powerful as they would otherwise be. In this way, the mother's soft parts are safeguarded against lacerations.

In cases of perineal lacerations, the anesthesia may be maintained until the sutures have been placed; but when no repair is necessary, the patient is allowed to come out, which she generally does a few minutes after the delivery.

This form of anesthesia has been followed by most men, except in those cases when it is contraindicated with uniform good results. In late years, the use of nitrous acid, oxygen-gas anesthesia has been popularized by some operators with good results, but this requires the services of an expert anesthetist and special apparatus. Whenever used, oxygen should be exhibited freely in order to prevent the child from being too deeply narcotized.

In recent years, as a result of agitation through the lay press and the moving picture, considerable interest has been aroused in a method of delivery know as "twilight sleep," or "Dämmerschlaf." That is a form of amnesia or loss of memory of recent events. This method had been tried out about fourteen years ago and was abandoned as a dangerous method of delivery by all except Kroenig and Gauss, but on account of the wide advertisement it received at the hands of the lay press, was tried out by a great many physicians, and, while most of them have abandoned it, some either because the laity demanded it, or because of their desire to be up-to-date in medicine would try any fad, continued its use, and judging from their reports almost all cases are amenable to this form of treatment. Their results are brilliant. So far as they are concerned, there is no danger to either mother or child. If the child is asphyxiated, it is due to some other cause. In short, their results have been very brilliant, but in the hands of such men as Williams, Edgar, De Lee, Hurst and Hare, the results have been rather disappointing, and, in some cases, disastrous. Even those who claim to be getting results give us the following instructions: The best patient is a well-educated woman of the upper class (financially), one who is open to suggestion is preferred. It should be conducted in a well-equipped maternity hospital by one who is expert in its administration. Then there should be at least two or three nurses within call; an assistant should be at hand. It takes five to manage an occasional delirious woman, even though she is of our decadent upper class. A darkened room, and instruments ready for immediate delivery. The very advocates of this method seem to be taking undue precautions. Why all this "safety first," if the method is so safe in their hands?

One of the main reasons for the use of this method that is advanced by the advocates of "twilight sleep" is that the women of the upper class have no children, or very few, on account of the fear of the pain in labor. Does the professional abortionist use "twilight sleep?" And you advocates of "twilight sleep" will agree that he thrives on the results of the marriages of our upper classes. It is not the pain at labor that deters our so-called upper classes to fight shy of pregnancy. It is the fact that with the advent of pregnancy all their social activities cease—they are social recluses—they cannot attend their card parties as usual. That is the thing that prevents our women of the upper classes from procreating. It is nothing but selfishness and the lack of the higher ethics and their appreciation of their duties to the race.

### THE INDICATIONS AND CONTRAINDICATIONS IN THE USE OF PITUITRIN IN OBSTETRICS.

Since November, 1909, when Bell published his results in a series of twelve cases in which he used the extract of the pituitary gland to stimulate the inert uterus to contractions, we have passed through a period of its use and abuse until we have reached a point where we can use it with comparative safety in selected cases.

When the large chemical houses began to commercialize the drug, every medical journal ran advertisements reading "discard forceps, use pituitrin, the new oxytocic"; and, as in their advertising of their vaccines, they influenced the mass of the medical profession to make use of a most dangerous drug in a great many cases with most disastrous results to both mother and child. But, through all this period of hysteria, it has finally earned a permanent place in the obstetric armamentarium. Pituitrin is a most valuable drug when used in properly selected cases—in the interest of the mother, not the doctor. In the hands of an unscrupulous or ignorant obstetrician, it is a most dangerous drug. It has its indications and contraindications, and for a drug that has only been in use seven years, they are very definite. The contraindications are more definite than the indications.

We must be most careful in the use of this drug in our primipara, as the future health of our young mother depends upon the conduct of her first pregnancy and labor. The physiological process that pregnancy and labor is, there is a remarkable number of complications which have to be guarded against. Lacerations and infectious head the list, and, if properly watched and its indications met, there would be a tremendous reduction of chronic invalidism in our best citizenship—the young mother. And if we as obstetricians would watch our cases more carefully, labor would not be the ordeal it is in many cases, and the cry of the lay press for "twilight sleep" would fall on deaf ears.

The dangers of the use of pituitrin to the mother are: ruptured uterus, more frequent cervical tears, too rapid delivery with laceration of the perineum. To the child: too early separation of the placenta with subsequent death of the fetus, due to the pains being too tumultuous.

The ideal safety zone for pituitrin would be the following case: Multiparae—who give a history of previous deliveries—full dilation—the soft parts relaxed—normal presentation, and the presenting part engaged. In this case ½ c.c. of pituitrin will give us the most brilliant results. For the sudden and tumultuous pains, ether analgesia may be employed. In a primipara, when the cervix is fully dilated, or almost so, the presenting part engaged, a 5-minim dose of pituitrin will often sensitize the uterus so that the normal stimuli will give us stronger contractions. When there is a slight obstacle, as in R. O. P., a tentative dose of pituitrin may be given, as we know that nothing tends to rotation as good strong pains, but, in these cases, we must be prepared to deliver. In the great class of cases, where low or even mid forceps is indicated, pituitrin may be given, with very gratifying results. In primary and secondary inertias not due to obstructions, pituitrin may be used.

In a Caesarean section, when we want strong contractions, we use pituitrin, followed by a hypodermatic of ergotol in order to maintain contractions.

Pituitrin should not be used in any malpositions—in twin pregnancies—in dyslochias of various kinds—in hydramnios—in patients who have had a Caesarean section, on account of the danger of rupture of the old scar. In marked contractions of the pelvis, pituitrin should not be used.

Pituitrin will not cause abortion, but when manual dilatation, or packing of the cervix is resorted to to bring on pains, pituitrin will aid and often one dose will suffice to terminate labor in three or four hours.

The dose of pituitrin is from 5 to 15 minims, with the consensus of opinion favoring the lesser dose.

